# Guided Bone Regeneration of an Atrophic Maxilla Using Heterologous Cortical Lamina

**DOI:** 10.1155/2019/5216362

**Published:** 2019-06-11

**Authors:** Carlos Polis-Yanes, Carla Cadenas-Sebastián, Patricia Gual-Vaqués, Raúl Ayuso-Montero, Antoni Marí-Roig, José López-López

**Affiliations:** ^1^School of Dentistry, University of Barcelona, University Campus of Bellvitge, Barcelona, Spain; ^2^Oral Health and Masticatory System Group (Bellvitge Biomedical Research Institute) IDIBELL, University of Barcelona, L'Hospitalet de Llobregat, Barcelona, Spain; ^3^Department of Maxillofacial Surgery, University Hospital of Bellvitge, Catalonia, Spain; ^4^Department of Odontostomatology, Faculty of Medicine and Health Sciences (Dentistry), Odontological Hospital University of Barcelona, University of Barcelona, Spain

## Abstract

Alloplastic dental implants are currently the best way to replace lost teeth. In order to achieve good function and prognosis of dental implants, having bone and soft tissue to support them is necessary. When the amount of bone left is not enough to ensure the outcome of the implant, techniques such as shorts implants, zygomatic implants, or guided bone regeneration have been used. Even though autologous bone is mostly the “gold standard,” other biomaterials such as xenografts have led to the reduction of the morbidity of treatments and to the improvement of the regeneration technique outcomes. We present a clinical case of severe atrophy of the maxilla in which we used different types of biomaterials: heterologous cortical lamina, xenograft and autologous bone, and microscrews.

## 1. Introduction

Teeth are necessary organs for the development of a normal life that take part in different functions such as the mastication, phonation, and maintenance of a functional orofacial anatomy. In the absence of the teeth, dental implants have proved to be efficient to replace lost teeth. In order to ensure the long-term function of dental implants, most studies confirm the importance of having and maintaining a good peri-implant bone and enough soft tissue (gingiva) [1].

When the amount of bone is not enough to place dentals implants, sometimes the professional has to use techniques of guided bone regeneration (GBR), bone distraction, sinus elevation technique, block grafts, or implant alternatives (short implants, zygomatic implants, etc.) [1]. Throughout the years, several techniques have been proposed to regenerate the alveolar bone. The type of bone defect or the prosthetic rehabilitation and the preferences of the clinician and the patient will lead the professional to choose one technique or the other. In terms of bone condition, the ideal material for bone regeneration should be osteoconductive, osteoinductive, and osteogenic. Autologous bone is still nowadays the gold standard because of its osteogenic property [2, 3]. Besides the bone condition, other bone properties that should be expected from a regeneration biomaterial are (i) osteoconduction; (ii) stimulation of neoangiogenesis; (iii) absence of antigenic, teratogenic, or carcinogenic reactions; (iv) boundless source; (v) satisfactory and stable structure; (vi) minimum morbidity and complications; (vii) hydrophilic nature; (viii) easy handling; and (ix) low cost [3].

GBR uses barrier membranes, including resorbable and nonresorbable membranes, in order to avoid certain types of nonosteogenic, rapidly proliferating cells, such as epithelial and connective tissue cells. And on the contrary, these barriers promote the growth of slow-maturing tissue made by osteoforming cells. These membranes are considered an essential part of the GBR treatment [3]. Among the different membrane systems and materials that have been proposed, any membrane should meet the following criteria: biocompatibility, integration by the soft tissues, clinical manageability, ability to isolate the bone graft, and adequate mechanical and physical properties [4, 5].

## 2. Objective

The aim of this study is to report a clinical case that deals with bone regeneration, using as a bone graft a mixture of autogenous and xenogenic bone and as a membrane a cortical bone lamina fixed with microscrews.

## 3. Case Presentation

**Figure 1:**
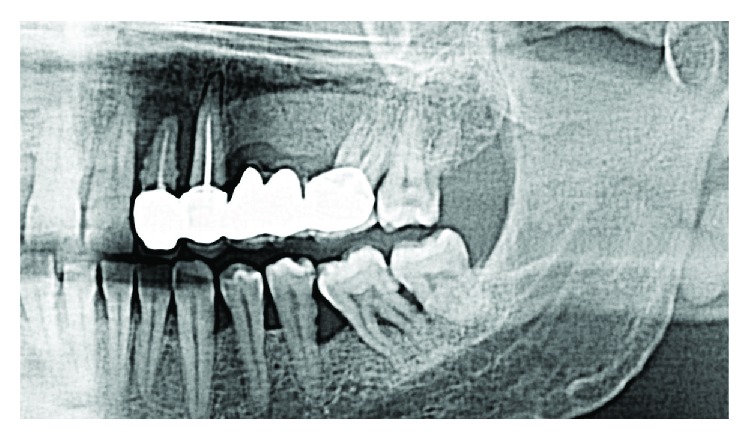
Orthopantomography previous to dental extractions.

**Figure 2:**
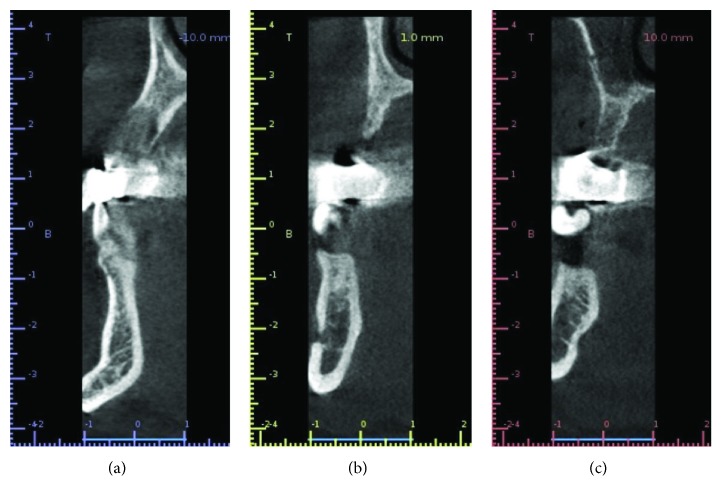
Previous CBCT.

A 45-year-old man presented mobility of a metal-ceramic fixed bridge in the second quadrant after ten years of function (Figure 1). After the exploration, the bridge and the pillar teeth were considered nonrestorable, and in the Cone Beam Computed Tomography (CBCT), a severe loss of the alveolar bone of the second quadrant is evidenced (Figure 2). Extraction of the teeth, regeneration of the lost bone, and following rehabilitation with dental implants were the agreed treatment.

**Figure 3:**
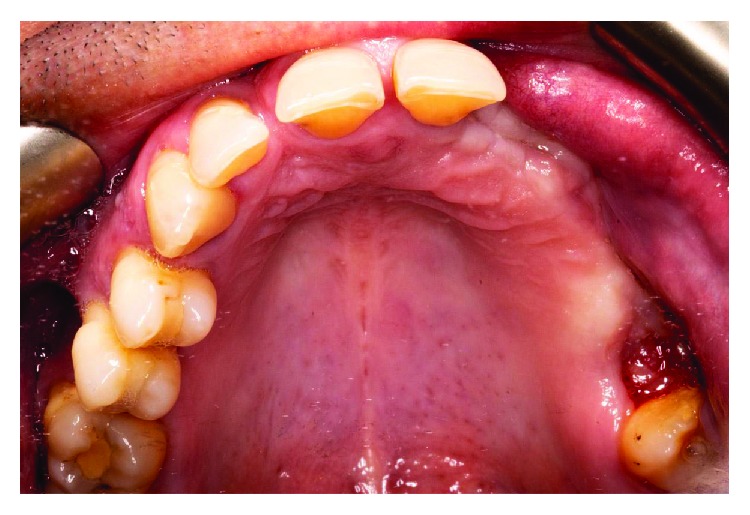
Intraoral view before guided bone regeneration surgery.

**Figure 4:**
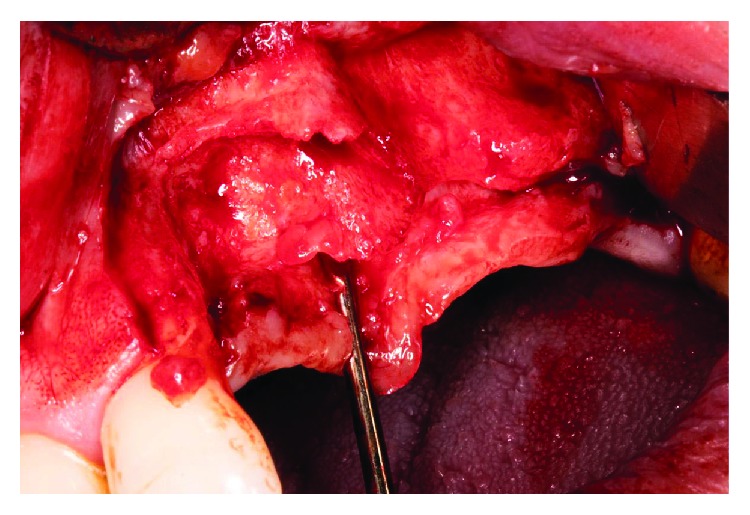
Appearance of the bone after the mucoperiosteal flap. There is a great defect with horizontal and vertical component, not suitable for the placement of dental implants.

**Figure 5:**
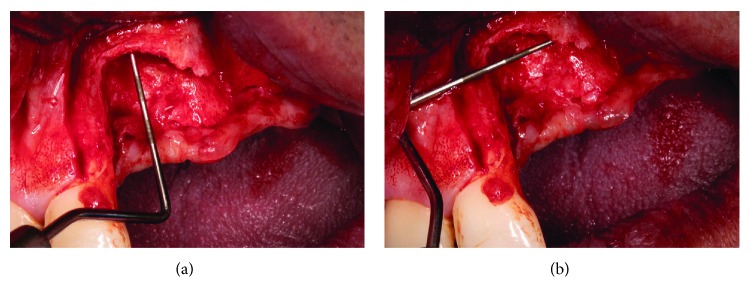
(a) Measurement of the most severe defect. (b) Measurement of the most severe defect.

**Figure 6:**
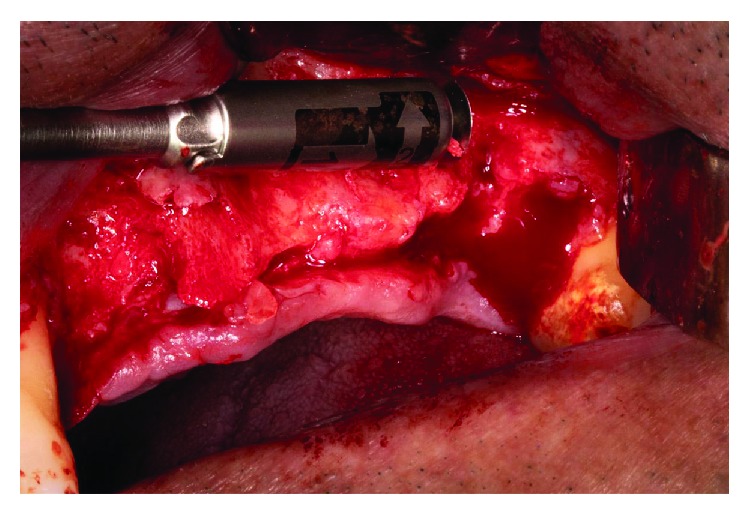
Bone scraper to decorticate and to collect autologous bone.

**Figure 7:**
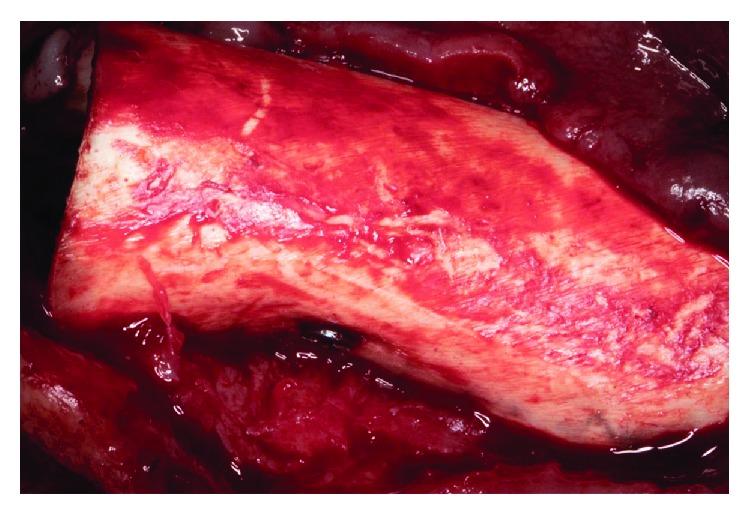
Cortical lamina fixed with microscrews in its palatal portion.

**Figure 8:**
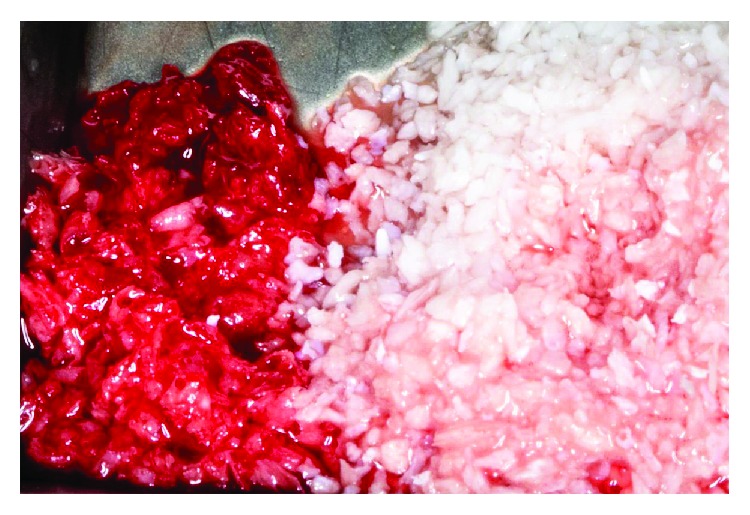
Autologous bone and xenograft.

**Figure 9:**
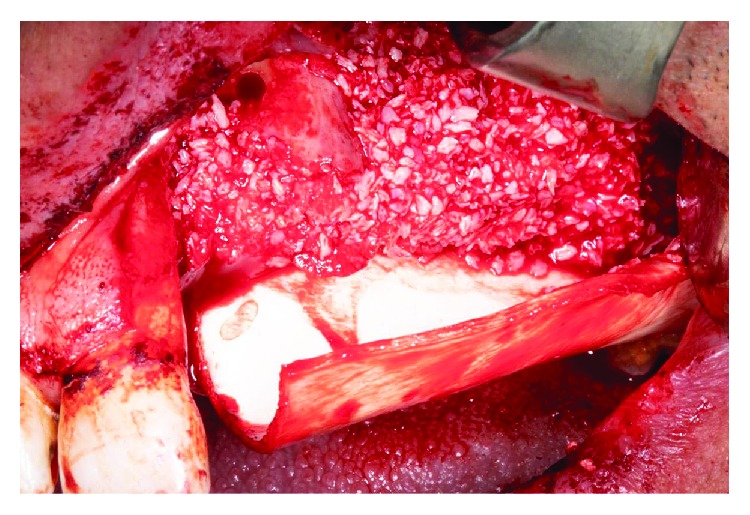
Bone graft placing taking as buccal limit the canine eminence conserved from the patient.

**Figure 10:**
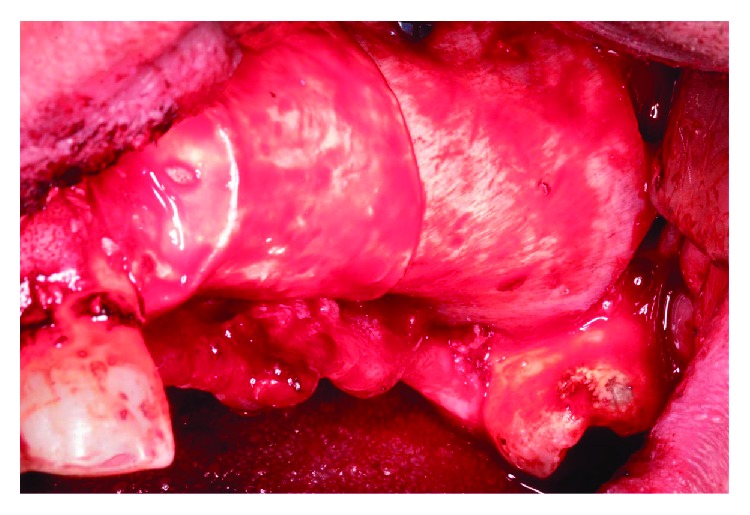
Buccal fixation of the cortical bone membrane with microscrews and covering of the mesial defect with a resorbable collagen membrane.

**Figure 11:**
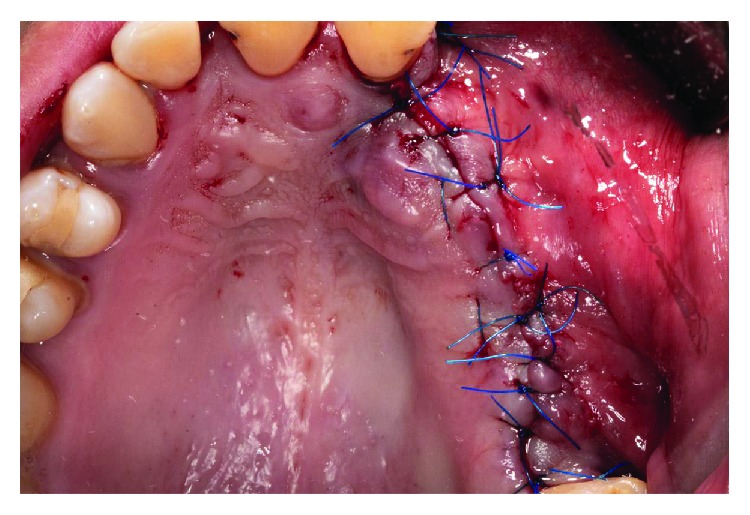
Suture without stress using monofilament suture.

**Figure 12:**
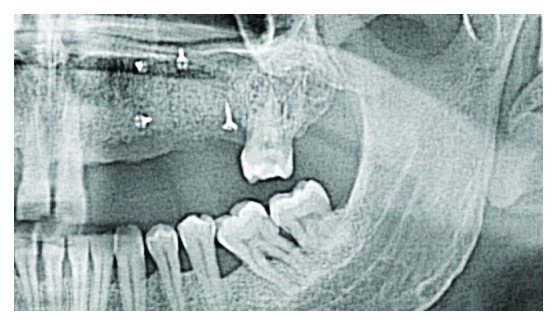
Orthopantomography after the surgery.

After the teeth extraction, we decided to wait a month to make sure the healing and stabilization of the soft tissues (Figure 3). In a second surgery stage, we performed a regenerative surgery. A heterologous cortical lamina (OsteoBiol Lamina® from Tecnoss®) was decided to be used instead of other barrier techniques, such as a titanium mesh, because of its resorbable condition. The surgical procedure was as follows: (i) mucoperiosteal flap with vertical discharges (Figures 4, 5(a), and 5(b)); (ii) periosteoplasty techniques; (iii) decorticalization and bone collection with a bone scraper (Figure 6); (iv) palatal fixation of the cortical lamina with two microscrews—no previously hydration is needed—(Figure 7); (v) filling of the defect with mixture of autologous bone and heterologous bone (OsteoBiol Apatos® from Tecnoss®) (Figures 8 and 9); (vi) vestibular fixation with two microscrews; (vii) mesial sealing with heterologous collagen membrane and resorbable polyglycolic acid suture (Serapid® from Serag-Wiessner®) (Figure 10); (viii) hydration with physiological serum prior to suture; and (ix) closure by first intention, without tensions, using monofilament suture, with simple and mattress stiches that relieve stress when inflamed (Figure 11). Immediately after the surgery, a control orthopantomography was taken (Figure 12).

The treatment was performed under antibiotic coverage with amoxicillin 750 mg (1 comp/8 h) 24 h before and 7 days later. 11.4 mg of postoperative intramuscular (gluteus) betamethasone was administered right after the surgery and dexketoprofen 25 mg (1 comp/8 h) was prescribed for 5 days. An antiseptic topical gel based on 0.2% chlorhexidine (one application every 8 hours) for 10 days was given to the patient. After 10 days, the suture was removed. During the bone healing period, the patient was told not to use any removable prostheses.

**Figure 13:**
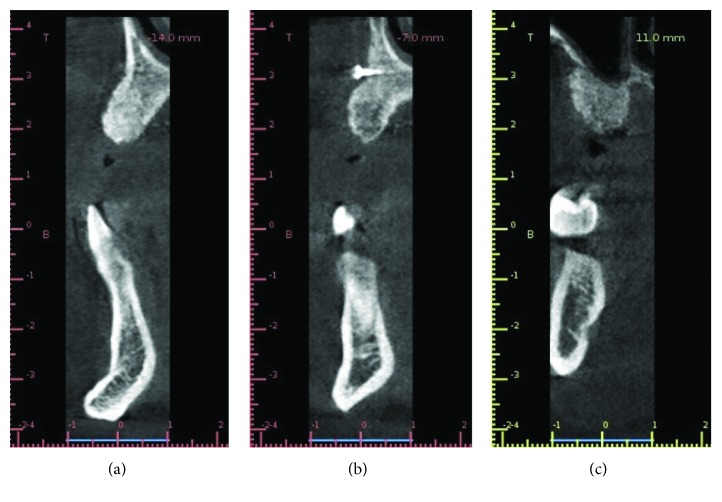
CBCT after healing during 6 months.

**Figure 14:**
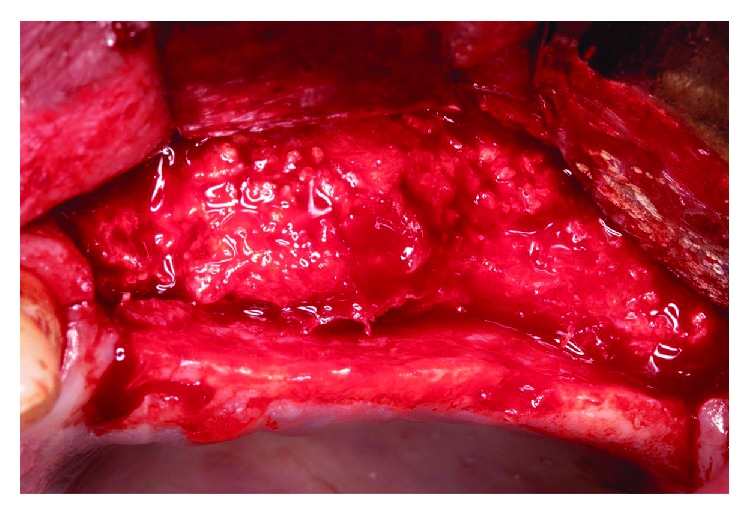
Clinical view after 6 months during dental implant placement.

**Figure 15:**
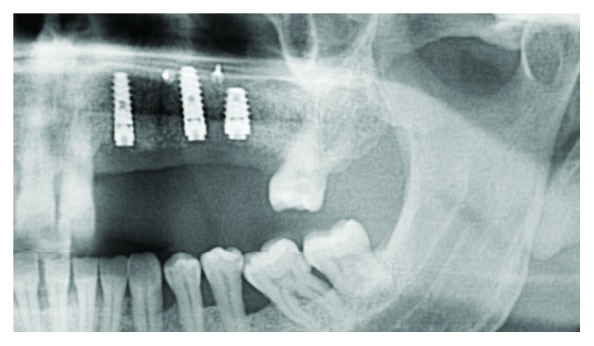
Orthopantomography after dental implant surgery.

Six months after the surgery, a new CBCT was performed (Figure 13) for implant planning and three internal conical connection implants (Galimplant® Sarria, Lugo, Spain) were placed in positions 22, 24, and 25 (3.5 × 12 mm, 4 × 12 mm, and 4.5 × 8 mm, respectively), with an insertion torque of 30 N/cm (Figures 14 and 15). During this surgery, the microscrews that blocked the implant placement were removed.

After four months, prosthetic rehabilitation was made by another clinician in another dental office, and thus, the follow-up was not possible.

## 4. Discussion

During the first 24 hours after regenerative surgery, the spaces are filled by a blood clot that is then resorbed by macrophages and neutrophils and replaced by granulation tissue rich in mesenchymal stem cells and blood vessels, allowing nutrients and cells to reach the site, forming the osteoid tissue [5]. Following this, there is a deposit of minerals and then bone tissue is formed, around which the bone continues to mature into lamellar bone. We will find bone neoformation about four weeks after the GBR. The main role of the membranes is to exclude connective and epithelial tissue cells from the area of the wound to be regenerated and also to create and maintain the space in which the pluripotent and osteogenic cells are free to migrate [5].

Currently, barrier membranes are considered necessary to carry out a successful GBR. Nevertheless, the potential hostage and cell activation of each membrane has not been established yet [4].

Elgali et al. [4] reported in their meta-analysis and systematic review soft tissue complications in approximately 16.8% of cases—with no significant differences between resorbable and nonresorbable membranes—concluding that the importance of the soft tissue management is essential to improve the prognosis of regenerative techniques. On the other hand, Soldatos et al. [5] concluded in their study the importance of the professional being familiar with the properties of the membranes to be used. For example, in case of height regeneration, the authors suggest the use of a nonresorbable membrane. The study concludes that the exposure of the membranes is a risk for the GBR and that the nonabsorbable membranes have a higher risk of exposure. Both membranes offer an adequate function as long as screws or the membrane itself is used to stabilize the GBR [5].

Titsinides et al. [3] concluded in their review that an ideal biomaterial for bone regeneration has not yet been developed. Nevertheless, predictable results are obtained with allografts, xenografts, and alloplastic grafts, all of them with their advantages and disadvantages [3]. The use of cortical autologous bone as a scaffold has been shown to be successful in a 10-year retrospective study by Khoury and Hanser [6] with 3328 patients treated with blocks for the management of atrophic bone ridges [6].

Bone blocks, cortical laminas, and membranes of heterologous cortical bone have been used successfully in recent years in plastic and maxillofacial surgery due to their plasticity and biocompatible structure and may be a less morbid alternative to distance block grafting. Being all of them resorbable is another advantage when compared to nonresorbable membranes and barriers [7, 8].

Regarding the use of cortical laminas, Lopez et al. [9] carried out GBR techniques with heterologous cortical lamina in twenty patients with thirty implants, twenty-four of them placed in the same surgery. They suggest the use of cortical laminas as a valid alternative to conventional GBR techniques. Similarly, Amr et al. [10] did a study on 14 patients who needed horizontal ridge regeneration. The sample was divided into two groups: group 1 underwent autologous block graft surgery while group 2 underwent heterologous cortical lamina surgery. Clinically and radiographically, there were no statistically significant differences between the two groups in terms of the bucco-lingual bone gain. The histomorphometric analysis showed no statistically significant differences between the mean areas of the bone surface in the two groups and no statistically significant difference between the mean osteoblast counts in the two groups. Thus, the authors concluded that xenogenic cortical lamina can be successfully used to increase the horizontal alveolar ridge as an alternative to the autogenous block bone graft.

Showing a different application for the cortical lamina, Scarano et al. [11] performed a randomized clinical study among twenty patients in which two different techniques of maxillary sinus floor elevation with lateral window were used. In one group, heterologous cortical lamina was used and the sinus cavity was not filled up with any biomaterial. In the second group, 100% porcine heterologous bone graft was used to fill up the sinus cavity plus a porcine heterologous collagen membrane to close the window. This study showed that the use of heterologous cortical laminas is a valid technique for the mechanical support of the sinus membrane. CBCT outcomes showed that the material was not completely resorbed after six months, although it was clearly integrated into the bone.

In another publication from the same authors, Scarano et al. [12] used the heterologous cortical lamina for the mechanical support of sinus membranes to preserve the space in sinus floor augmentation and showed the importance of Cone Beam Computed Tomography to evaluate the efficacy of this GBR technique.

## 5. Conclusion

Heterologous cortical laminas used as barrier membranes are a plausible biomaterial to be used in GBR, especially in medium and large bone defects. Long-term randomized studies are necessary to compare cortical lamina properties with other types of membranes that are more commercialized and well-studied.
